# Targeting altered Nme heterooligomerization in disease?

**DOI:** 10.18632/oncotarget.22716

**Published:** 2017-11-27

**Authors:** Issam H. Abu-Taha, Christiane Vettel, Thomas Wieland

**Affiliations:** ^1^ Institute of Experimental and Clinical Pharmacology and Toxicology, Medical Faculty Mannheim, Heidelberg University, Mannheim, Germany; DZHK (German Center for Cardiovascular Research), Partner Site, Heidelberg-Mannheim, Germany

**Keywords:** Nme, Nm23, cancer metastasis, heart failure, myocardial contraction

The enzymatic activity of nucleoside diphosphate kinases (NDPK), which are encoded by members of the *nme* gene family, has been discovered in the 1950s. It removes the terminal phosphate from a nucleoside triphosphate (NTP) and adds it to a nucleoside diphosphate (NDP) and thus it is important for nucleotide homeostasis in every living cell. At least the four group I Nme proteins (Nme1 – Nme4) carry this enzymatic activity. Nme1 and Nme2 are most abundant and ubiquitously expressed. In the 1990s it became evident that enhanced cancer metastasis was linked to reduced expression of Nme1. Metastasis, the colonization of distant sites by a tumor, is often more life threatening than the growth of the primary tumor, which can be surgically removed in many cases. Thus, the identification of the function of Nme1 underlying its metastasis suppressor activity is a long standing goal in research [1].

**Figure 1:**
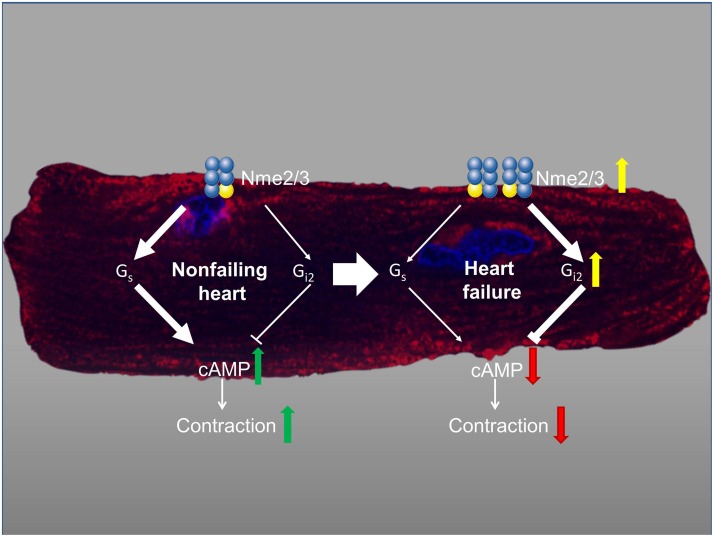
Schematic view of the switch from predominantly Nme2(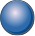)/Nme3(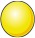)-mediated G_s_ signaling in non-failing heart to G_i_-dominated signaling in heart failure The cardiomyocyte in the back highlights the distribution of Nme3 (stained in red) after stimulation with isoproterenol. Nuclei are stained in blue. For details see [5].

It turned out that Nmes are “sticky” proteins. They obviously are not only found in many protein complexes but also bind to DNA and lipids. Thus a huge variety of functions have been attributed to Nme proteins, ranging from NTP supply, protein histidine kinase activity, exonuclease activity on DNA and protein scaffold as well as scavenger roles [2]. Therefore, the unique and to be targeted function of the Nme1 metastasis suppressor most likely does not exist. Recent work on Nme2 and Nme3 shed however light on an aspect nearly neglected so far in Nme research, heteroligomerization. Eukaryotic Nmes of the class I family form hexamers consistent of six enzymatically active monomers. Both, homo- and heterohexamers have been described [3]. Previous work from our laboratory and others linked Nme2 to heterotrimeric G protein activation, again acting as NDPK for GTP supply, as protein histidine kinase on the G protein β-subunit and as a scaffold organizing G protein-mediated signal transduction in caveolae [4]. Recently, we established that the complex formation with the G proteins G_s_ and G_i_ Nme2/Nme3 requires heterooligomerization [5]. In cardiomyocytes, the Nme2/Nme3 oligomers obviously regulate not only the activation of G proteins but also their abundance at the plasma membrane and their accessibility by G protein - coupled receptors. Interestingly, the preference of the interaction of the Nme2/Nme3 complexes with the G proteins switches during the development of end-stage heart failure, a condition, at which the expression of Nme3 and G_i_ proteins is additionally upregulated. Whereas in healthy human heart Nme2/Nme3 oligomers are preferentially bound to G_s_, in end-stage heart failure G_i2_ was found predominantly in the complex. This switch has profound consequences on the resulting signal transduction (Figure 1). An increased complex formation of Nme2/Nme3 with G_s_ increases cAMP formation and contractility. In contrast, if G_i2_ is bound, cAMP formation is constitutively suppressed which is in accordance with the observations from failing human hearts. Taken together, the data showed that the composition and localization of G protein signaling complexes can change over time during disease development and in response to altered Nme heteroligomerization.

As Nme1 has also been shown to form heterooligomers at least with Nme2 [3], it is worthwhile to speculate that the composition of this heterohexamers has influence on the interaction and localization properties and is thus involved in the multifaceted actions also of this protein. As for Nme2, the exact composition of the Nme1 interactome under specific experimental and/or clinical conditions is still unknown. It can however be expected that the advances in super-resolution live-cell imaging methodologies will help to elucidate the subcellular localization of signaling complexes and their organization in near future. Insights into the temporal dynamics of the Nme oligomer composition and their interactome will certainly help to understand the function of this enzyme in greater detail and offering possibilities to interfere with or enhance specific pathways.

So far only rather unspecific pharmacological inhibitors of the NDPK activity such as ellagic acid are available and no Nme - subtype specific modulators have been reported [6]. Therefore, we are still far off from translation into therapy. With regard to the protein histidine kinase activity of Nme2, however, some promising data are available. A counteracting histidine phosphatase named PHP (or PHPT-1) has been discovered in 2002 [7]. In cellular models, it has meanwhile been proven that histidine phosphorylation of the intermediate conductance potassium channel K_Ca_3.1 by Nme2 is required for full activation of the channel and this activation can be reversed by local application of PHP. As the Nme2-induced K_Ca_3.1 activity in vascular smooth muscle as well as in inflammatory T-cells is required for smooth muscle cell proliferation and as well as cytokine release from T-cells, like K_Ca_3.1 deficient mice, Nme2 depleted mice are protect from deleterious neointima formation in vessels as well as severe colitis in a model of inflammatory bowel disease [8]. Thus, a targeted delivery of the rather small (~15 kDa) PHP to inflammatory or vascular smooth muscle cells would raise the possibility to locally inhibit the deleterious K_Ca_3.1 activation. Peptides or small molecules interfering with the specific interaction sites of Nme2 with K_Ca_3.1, which might also require heterooligomerization with another Nme to gain specifity, could offer the most promising modulators for a future therapeutic approach. Taking into account that Nme4 has a rather specific function in mitochondria, heterooligomers of Nme1, Nme2 and Nme3 and their interactome are the ones to be studied in greater detail in the future.
